# Supplementary material for: In vitro reconstitution of Sgk3 activation by phosphatidylinositol 3-phosphate
Source: J Biol Chem. 2021 Jun 25;297(2):100919. doi: 10.1016/j.jbc.2021.100919 (PMC8318898; doi:10.1016/j.jbc.2021.100919)
Supplement: Figures S1–S4 and Table S1 [file mmc2.pdf]

In vitro reconstitution of Sgk3 activation by phosphatidylinositol-3-phosphate

Daniel Pokorny[1,2], Linda Truebestein[1,2], Kaelin D. Fleming[3], John E. Burke[3,4] and Thomas A. Leonard[1,2,5*]

Supporting Information

Materials included:

Figures S1-S3

S1. Characterization of recombinant Sgk3.

S2. Sgk3 is specifically and allosterically activated by PI3P.

S3. Preparation of FITC-labeled Sgk3PX for liposome pelleting assays.

Tables S1-S3

S1. Statistics for HDX datasets associated with this manuscript.

S2. Sgk3 peptides identified by tandem mass spectrometry.

S3. Phosphorylated peptides with ambiguous site localization.

Fig. S1. Characterization of recombinant Sgk3.

A

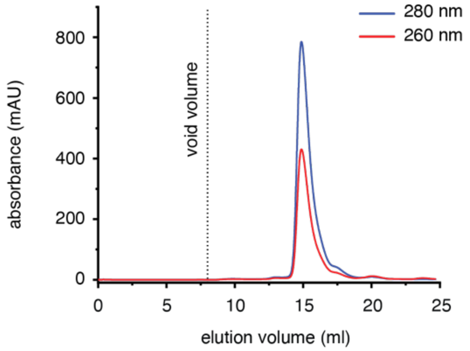

B

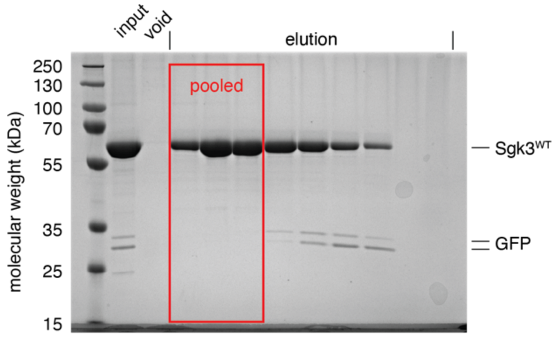

C

GluC digest (coverage = 48.4%; 55.7% coverage of kinase domain)
MS/MS of Sgk3 purified from Sf9 insect cells

Residue #	Location	ratio mod/base (fraction modified)
21	PX domain	∞ (no base peptide detected)
144	PX-kinase linker	0.42 (0.30)
320	activation loop	0.27 (0.21)
461	turn motif	0 (no modified peptide detected)
486	hydrophobic motif	0 (no modified peptide detected)

D

GluC digest (coverage = 68.7%; 76.8% coverage of kinase domain)
MS/MS of Sgk3 purified from HEK293 cells

Residue #	Location	ratio mod/base (fraction modified)
30	PX domain	0.02 (0.02)
320	activation loop	0.06 (0.06)
461	turn motif	0 (no modified peptide detected)
486	hydrophobic motif	0 (no modified peptide detected)

E

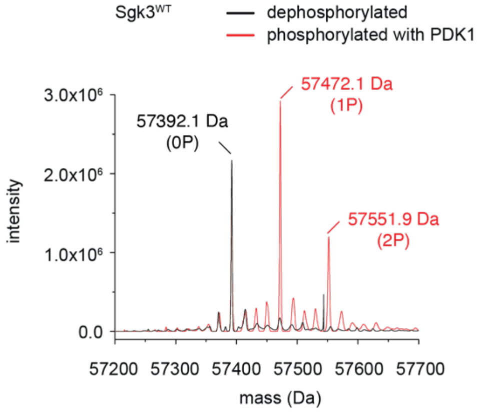

Mass (Da) (expected)	Mass (Da) (observed)	Δmass (Da)	Matching modifications
57391.8	57392.1	+0.3	none
	57392.1	+0.3	none
	57472.1	+80.3	1P
	57551.9	+160.1	2P

F

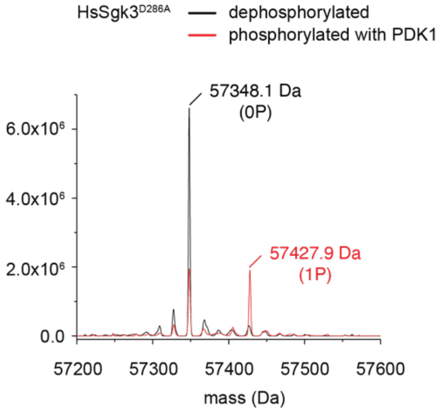

Mass (Da) (expected)	Mass (Da) (observed)	Δmass (Da)	Matching modifications
57347.8	57348.1	+0.3	none
	57348.1	+0.3	none
	57427.9	+80.1	1P

G

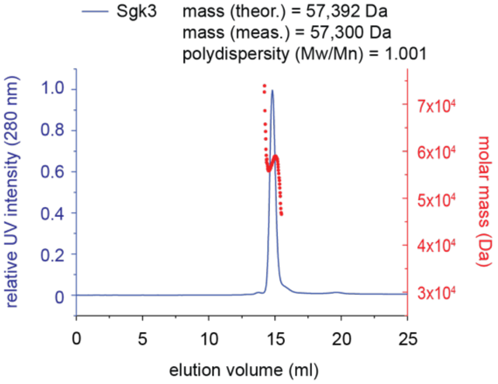

H

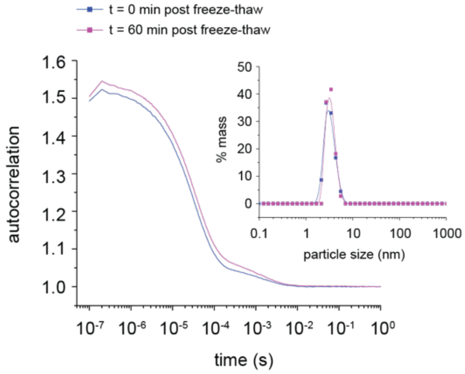

Fig. S1. Characterization of recombinant Sgk3.

A) Size exclusion chromatography of $Sgk3^{WT}$ purified from Sf9 insect cells.

B) Representative gel indicating Sgk3 purity and separation from cleaved eGFP. Pooled fractions used for all downstream biochemistry are indicated with the red box.

C) GluC digest MS/MS phosphomapping of $Sgk3^{WT}$ purified from Sf9 insect cells.

D) GluC digest MS/MS phosphomapping of $Sgk3^{WT}$ purified from transiently transfected HEK293T cells.

E) Intact MS of dephosphorylated (black) and PDK1-phosphorylated (red) $Sgk3^{WT}$ protein.

F) Intact MS of dephosphorylated (black) and PDK1-phosphorylated (red) $Sgk3^{D286A}$ protein.

G) SEC-MALS profile of $Sgk3^{WT}$ indicating molecular weight and monodispersity.

H) DLS measurement of $Sgk3^{WT}$ in conditions of kinase assays measured after thawing (blue curve) and after 60 min incubation at RT (pink curve). Particle size distribution remains unchanged after 60 min incubation.

Fig. S2. Sgk3 is specifically and allosterically activated by PI3P.

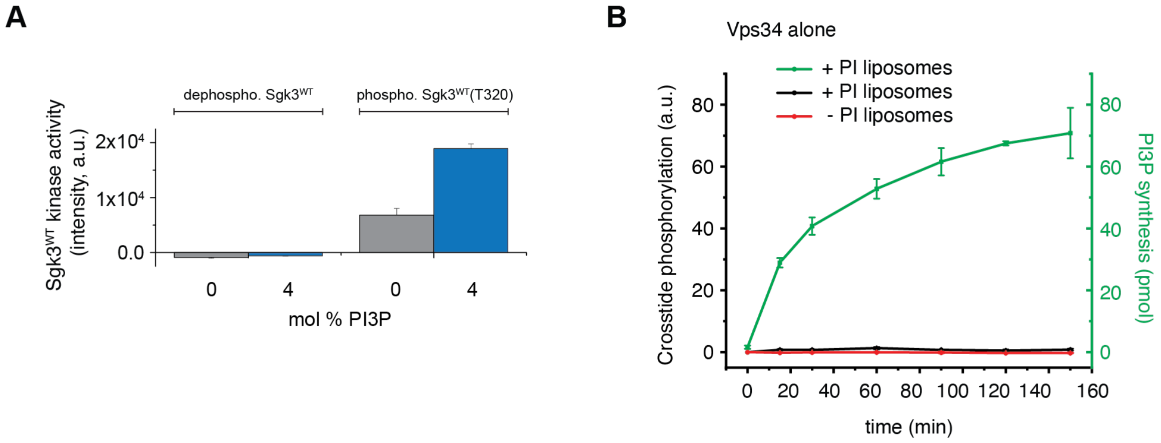

Fig. S2. Sgk3 is specifically and allosterically activated by PI3P.

A) Kinase activity of dephosphorylated Sgk3^{WT} vs. PDK1-phosphorylated Sgk3^{WT} in presence of 100 μM Crosstide with 0% PI3P (grey bars) or 4% PI3P (blue bars). Error bars are the standard deviation of three independent experiments.

B) Vps34 control for non-specific Crosstide phosphorylation. Phosphorylation of Crosstide by Vps34 was measured in absence of Sgk3 and in presence (black line) or absence (red line) of PI liposomes. Generation of PI3P by Vps34 was simultaneously measured (green line). Error bars are the standard deviation of three independent experiments.

Fig. S3. Preparation of FITC-labeled Sgk3^{PX} for liposome pelleting assays.

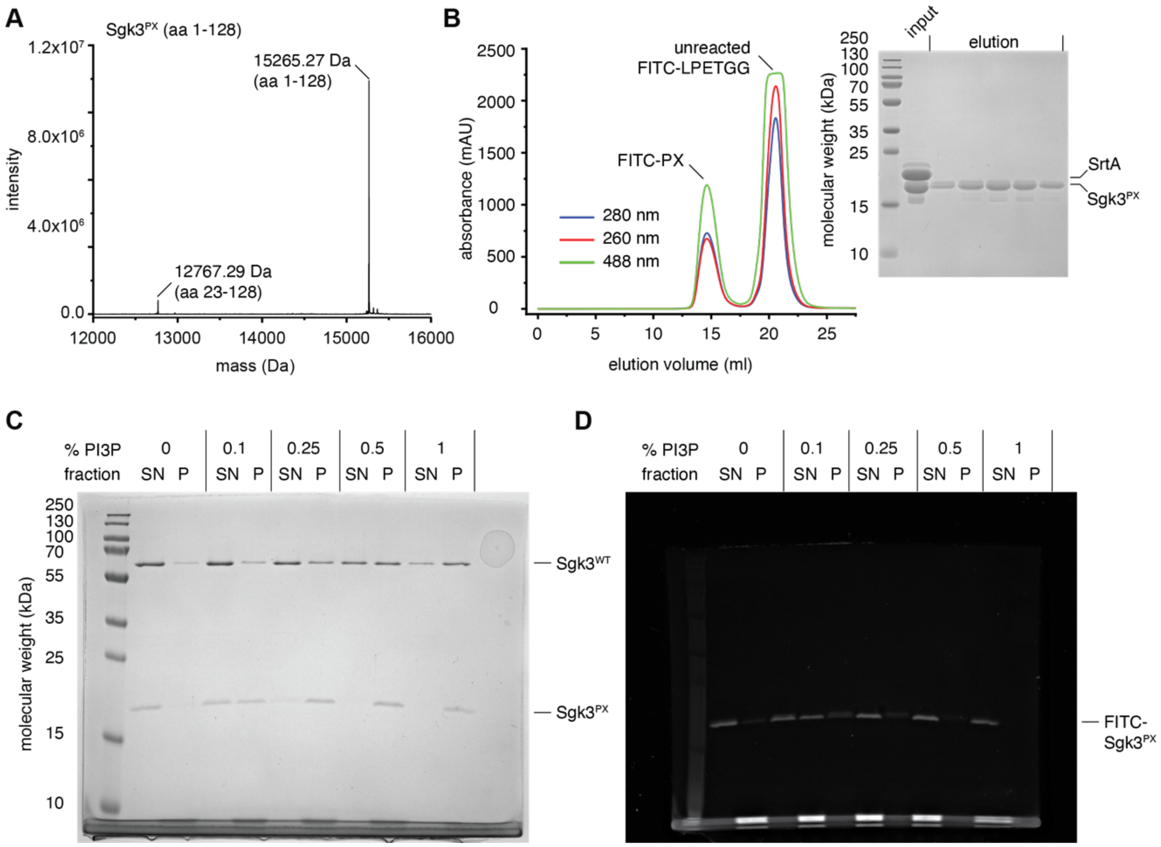

Fig. S3. Preparation of FITC-labeled Sgk3PX for liposome pelleting assays.

A) Intact mass spectrum for Sgk3^{PX}. A small amount of an N-terminal degradation product was observed.

B) Sortase labeling of the PX domain. His-tagged Sortase was removed with a NiNTA column prior to size exclusion chromatography on a Superdex 75 10/300 column to separate FITC-labeled PX domain from unreacted FITC peptide.

C) Raw data for liposome pelleting assay quantified in Fig. 3A. Coomassie-stained gel containing supernatant (SN) and pellet (P) fractions. $Sgk3^{WT}$ and $Sgk3^{PX}$ are indicated.

D) Raw data for liposome pelleting assay quantified in Fig. 3A. Gel containing supernatant (SN) and pellet (P) fractions was imaged at 488 nm using a fluorescent imager. The FITC-labeled $Sgk3^{PX}$ domain is indicated. The gel images in panels C and D are derived from the same gel, imaged at 488 nm (panel D) prior to Coomassie staining (panel C).

Table S1. Statistics for HDX datasets associated with this manuscript.

**Data set**	**FL Sgk3 0% PI3P**	**FL Sgk3 5% PI3P**	**PX Sgk3 0% PI3P**	**PX Sgk3 5% PI3P**
HDX reaction details	$\%D_2O$=81.5% $pH_{(read)}$=7.5 Temp=18ºC	$\%D_2O$=81.5% $pH_{(read)}$=7.5 Temp=18ºC	$\%D_2O$=81.5% $pH_{(read)}$=7.5 Temp=18ºC	$\%D_2O$=81.5% $pH_{(read)}$=7.5 Temp=18ºC
HDX time course (seconds)	3, 30, 300, 3000	3, 30, 300, 3000	3, 30, 300, 3000	3, 30, 300, 3000
HDX controls	N/A	N/A	N/A	N/A
Back-exchange	Corrected based on $\%D_2O$	Corrected based on $\%D_2O$	Corrected based on $\%D_2O$	Corrected based on $\%D_2O$
Number of peptides	110	110	25	25
Sequence coverage	94.6%	94.6%	83.9%	83.9%
Average peptide /redundancy	Length=13.9 Redundancy= 3.1	Length=13.9 Redundancy= 3.1	Length=12.2 Redundancy= 3.1	Length=12.2 Redundancy= 3.1
Replicates	3	3	3	3
Repeatability	Average StDev=1.9%	Average StDev=2.1%	Average StDev=1.6%	Average StDev=1.1%
Significant differences in HDX	>7% and >0.5 Da and unpaired t-test ≤0.01	>7% and >0.5 Da and unpaired t-test ≤0.01	>7% and >0.5 Da and unpaired t-test ≤0.01	>7% and >0.5 Da and unpaired t-test ≤0.01
